# Channel Selection in Uncoordinated IEEE 802.11 Networks Using Graph Coloring

**DOI:** 10.3390/s23135932

**Published:** 2023-06-26

**Authors:** Jose Manuel Gimenez-Guzman, Ivan Marsa-Maestre, Enrique de la Hoz, David Orden, David Herranz-Oliveros

**Affiliations:** 1 Universitat Politècnica de València, Departamento de Comunicaciones, 46022 València, Spain; 2 Universidad de Alcalá, Computer Engineering Department, 28805 Alcalá de Henares, Spain; ivan.marsa@uah.es (I.M.-M.); enrique.delahoz@uah.es (E.d.l.H.); david.herranz@uah.es (D.H.-O.); 3 Universidad de Alcalá, Department of Physics and Mathematics, 28805 Alcalá de Henares, Spain; david.orden@uah.es

**Keywords:** graph coloring, channel assignment, IEEE 802.11

## Abstract

One of the big challenges in decentralized Wi-Fi networks is how to select channels for the different access points (APs) and their associated stations (STAs) in order to minimize interference and hence maximize throughput. Interestingly enough, de facto standards in terms of uncoordinated channel selection are quite simple, and in many cases result in fairly suboptimal channel allocations. Here, we explore how graph coloring can be used to evaluate and inform decisions on Wi-Fi channel selection in uncoordinated settings. Graph coloring, in its most basic form, is a classic mathematical problem where colors have to be assigned to nodes in a graph while avoiding assigning the same color to adjacent nodes. In this paper, we modeled Wi-Fi uncoordinated channel selection as a graph coloring problem and evaluated the performance of different uncoordinated channel selection techniques in a set of representative scenarios of residential buildings. The results confirm some of the widely accepted consensus regarding uncoordinated channel selection but also provide some new insights. For instance, in some settings, it would be better to delegate the decision on which channel to use to transmit the STAs, rather than having the AP make the decision on its own, which is the usual way.

## 1. Introduction

Perhaps the most pervasive scenarios in networking today are uncoordinated Wi-Fi networks. These are the kind of networks we can find in residential buildings, malls, and historical city centers. In these networks, also called infrastructure mode Wi-Fi networks, there are a number of Wi-Fi access points (APs) belonging to different parties (e.g., small business owners, house owners, tenants…). Each of these access points provides Wi-Fi connectivity to a set of client stations (STAs), which may be static (e.g., a Smart TV in a house) or dynamic (e.g., customers at a coffee shop). All these access points and stations communicate using the same shared *unlicensed spectrum* bands (typically 2.4 GHz, 5 GHz and, more recently, 6 GHz). Due to the pervasiveness of these scenarios, these frequency bands tend to be quite crowded in terms of transmission interference. Therefore, choosing the right frequency to operate can make a great difference in terms of performance and user experience.

The Wi-Fi spectrum is divided into channels (e.g., 11 channels in the US 2.4 GHz band), which typically overlap. Thus, a transmitting device (AP or STA) operating in a channel will suffer interference not only from other transmissions in the same channel but also from other transmissions in neighboring channels. The interference reduces as channels move “further away” in the frequency domain, being even negligible if the channels are apart enough. For instance, in the US 2.4 GHz band, channels 1, 6, and 11 do not interfere with each other and they are normally dubbed as “orthogonal channels”.

In coordinated Wi-Fi deployments, such as enterprise buildings, channel allocation for optimal operation is more or less straightforward: It is enough to deploy APs in strategic places so that the whole area of, e.g., the building can be covered using orthogonal channels in a way so that no APs that are in range are using the same channel. Even if such planning is not possible, centralized AP management allows for effective channel assignment optimization using centralized techniques [1], so that interferences are avoided and throughput can be maximized.

In decentralized Wi-Fi networks, however, optimal channel selection for the different APs and their associated STAs can be quite a challenge since there is no prior agreement among the different APs, and coordination emerges in a decentralized manner, using different techniques, such as [2]. Interestingly enough, *de facto* standards are quite simple, and, in many cases, result in fairly suboptimal channel allocations [1].

In this article, we explore the applicability of a well-known mathematical problem called *graph coloring* to model uncoordinated Wi-Fi networks and to inform the decisions about channel selection. In the classic version of graph coloring, we have the nodes (or vertices) and links (or edges) of a graph, and the goal is to “color” the graph, that is, to assign a color to each node so that no adjacent (linked) nodes receive the same color. The goal, in the most basic version, is to use the least possible number of colors, but there are variations of the problem. In particular, we proposed in [3] the *Threshold Spectrum Coloring (TSC)*, where the spectrum of available colors is limited and colors are endowed with an *interference matrix*. The goal of TSC is to arrive at a coloring using the available spectrum that minimizes interference. As the reader has probably deduced, this problem maps directly to the Wi-Fi channel assignment, so it will guide our discussion throughout the rest of this article.

When dealing with wireless network optimization, it is common to use models based on Markov processes, Petri nets, or graphs (such as the one we use here) in the early and middle stages of research, prior to implementation, before introducing more computationally costly tools, such as discrete event simulation. Our graph coloring model [4] is complementary to simulation models [5], providing the opportunity for researchers to compare different proposals with a lower computation cost than discrete event simulators. Of course, our model focuses on some of the features of the wireless networks while capturing others with less detail.

The main contributions of this paper are:

- We show a graph model that is well-suited to test and evaluate the distributed channel selection techniques (Section 4.1).
- We propose and implement several channel selection techniques, modeling them as a graph coloring problem. The main requirement of these techniques is that they are based on simple and easily measurable parameters from the nodes’ point of view, such as the interference level or the number of beacon frames received (Section 4.2).
- We demonstrate that the techniques based on the measurement of the interferences outperform those based on measuring the number of beacon frames (Section 5.2 and Section 5.3).
- We show that, although the use of non-orthogonal channels is one of the main features of IEEE 802.11 networks in the 2.4 GHz frequency band, the best channel selection techniques mainly use orthogonal channels (Section 5.4).

The rest of this paper is organized as follows. We first describe some of the most prominent works related to our proposal. Later, we briefly discuss the relationship between graph coloring and Wi-Fi channel selection (Section 3). Then, we detail the model we have used to evaluate channel allocations in a paradigmatic Wi-Fi deployment setting: residential buildings (Section 4.1). Finally, we compare the most relevant channel selection techniques (described in Section 4.2) and discuss the results (Section 5). Our results confirm some of the widely accepted beliefs regarding uncoordinated channel selection, which had not been formally studied so far, and, in addition, provide some new insights, summarized in Section 6.

## 2. Related Work

Channel selection in IEEE 802.11 networks has been a thoroughly studied topic during recent years due to its relevance for network performance and its complexity. One of the most prominent revisions of the research related to channel selection in a WLAN is [6]. However, it is already quite an old survey and there have been a number of proposals in this field since its publication. To classify the proposals, we can divide the channel selection techniques into two main types: centralized [1,3,7,8,9,10,11,12,13,14,15,16,17] or distributed [2,10,18,19,20,21,22]. It is important to highlight that the use of one type or another may depend on the kind of Wi-Fi network under consideration. For Wi-Fi deployments, where each AP is managed individually, we must resort to distributed or uncoordinated techniques. A well-known example of this type of scenario is a residential environment. However, in Wi-Fi settings that are managed by a central controller entity (such as a corporate network), we can make use of either centralized (coordinated) or distributed techniques, although typically the first option is chosen, as it is able to obtain higher performance if we can coordinate the different APs in the network. Simultaneously, channel selection techniques can also be classified into two main types: optimization approaches [1,7,8,9,13,14,18] and heuristic processes [2,3,10,11,12,15,16,17,19,20,21,22].

Now the different types of channel selection techniques have been categorized, we can now make a brief description of them. The number of approaches to channel selection techniques based on optimization processes is more limited. For example, in [7], the authors used integer linear programming to obtain an optimal centralized channel assignment, but it is restricted to three orthogonal channels and only considers interferences from APs, neglecting interferences from the other STAs. In [1], the authors proposed a centralized optimization technique based on an evolutionary-type algorithm called coral reef optimization with a substrate layer, which simulates some processes that really occur in living coral populations. This work considers the whole range of available channels in Wi-Fi, in the band of 2.4 GHz (as opposed to the works only considering orthogonal channels), and also takes into account interferences from the STAs. It is worth mentioning that the results are compared with a previously published manuscript [8] that uses simulated annealing to determine the optimal channel assignment. The authors in [9] proposed an optimization approach based on deep learning whose objective is to minimize the cochannel interference by focusing on the interferences from the APs. Finally, it is important to mention [18], as it considers an optimization approach suited for distributed Wi-Fi settings. In this work, the authors considered the problem of channel selection as a negotiation process and used belief propagation to address it, which is the first and unique gossip-like technique used in the context of channel selection in Wi-Fi networks.

Now the main work related to the optimization approaches has been described, we can now focus on the heuristic proposals. A discussion about the differences between the optimization-based approaches and heuristics as techniques for channel selection can be found in [23]. Notwithstanding, and without any doubt, the most prominent work and widely-used technique for channel selection is the one described in [2]. This technique, called *Least Congested Channel Search*, involves measuring the number of STAs using each channel and using the channel where this number is the lowest. Another interesting work is [10], where the authors proposed a two-phase radio resource management framework, with a first phase of channel assignment and a second one of user association for improving load balancing.

In Table 1, we show a summary of the main approaches for channel selection in Wi-Fi networks, showing whether they are centralized or distributed and whether they propose a centralized or heuristic approach. We also show whether those studies considered or not the cochannel interference, i.e., if they considered the partial overlap between the nearby IEEE 802.11 channels. From the table, we can situate our proposal within the related work. As can be seen, the proposal of distributed and heuristic approaches considering cochannel interferences has not received much attention from the scientific community. In addition, we impose on our proposals the requirement to keep the techniques as simple as possible and to use easily measurable parameters to inform the decision-making.

## 3. Graphs and Channel Selection in Wi-Fi

Graphs, defined as a set of nodes (vertices) and a set of links (edges) that connect some of those vertices, constitute a very useful tool to represent networks. The application of graphs has received thorough attention from the scientific community since its application is open to a wide number of research areas, with graph neural networks being a recent hot topic [24,25]. Additionally, graph coloring represents another hot topic in the field of discrete mathematics, attracting the interest of the scientific community from the fields of both engineering and mathematics, due to its theoretical challenges and applications [26]. One of the most outstanding applications of graph coloring is the frequency assignment problem [27] since assigning colors to the vertices of a graph is equivalent to assigning frequencies to the different elements composing a network.

**Figure 1:**
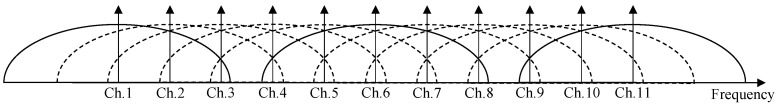
Arrangement of IEEE 802.11 channels in the 2.4 GHz frequency band in USA.

Although graph coloring is a thoroughly studied problem in discrete mathematics, the most common proposals are focused on issues such as avoiding monochromatic edges (i.e., no edge can connect two vertices of the same color). However, our problem of selecting channels for APs is different since we are interested in selecting channels in such a way that the STAs achieve a high throughput. This introduces key changes to the classic problem. One of the most challenging differences is the fact that non-monochromatic edges may also have a negative impact on the overall goodness of a solution. This is due to a peculiarity of the Wi-Fi spectrum, which is the fact that channels partially overlap. For instance, from the 11 channels that can be used in the 2.4 GHz frequency band in the USA, the set of channels that do not overlap is limited to three (called orthogonal channels: 1, 6, and 11) as shown in Figure 1. Interestingly enough, many of the proposals for channel selection in the literature [6] do not address the issue of adjacent channel interference, despite its impact on performance, which we have discussed in [28].

**Figure 2:**
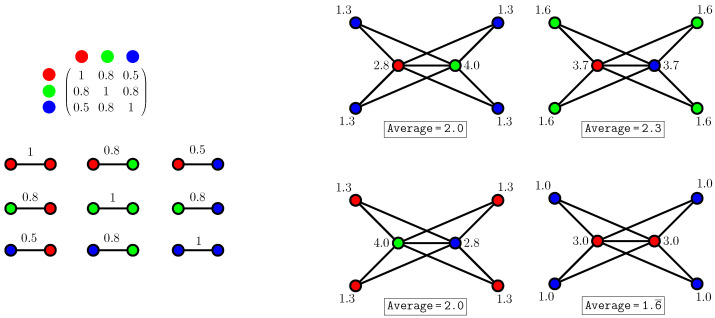
(**Left**): Interference matrix. (**Right**): All the possible colorings avoiding monochromatic edges and a better coloring allowing them.

Due to the cochannel interference between the adjacent channels, for an AP to select a channel, we should take into account the “distance” between the channels in the spectrum, as the “nearby” channels partially overlap in IEEE 802.11. This is why we proposed in [3] a type of graph coloring called *spectrum graph coloring* that is suitable for Wi-Fi channel selection. The goal of spectrum graph coloring is to find a coloring that minimizes interference according to a given interference matrix. In the Wi-Fi setting, the interference matrix specifies the effect that an AP operating in a channel has on the rest of the channels. An interesting result of this previous work is that optimal colorings may include monochromatic edges, which previous coloring conceptions forbade. An example of how graph coloring can be used to assign channels in a network where those channels partially overlap is shown in Figure 2, where we also show that avoiding monochromatic edges does not mean that interferences are minimized. Figure 2, on the left side, shows the possible interferences between pairs of APs depending on the channels (colors) assigned to them. In Figure 2, on the right side, the total interference at a vertex is computed as the sum of the interferences of its incident edges. We can see that when aiming to color the graph while avoiding the monochromatic edges, three options arise for the horizontal edge, each of them forcing the remaining color at the rest of vertices; these are the three subfigures of Figure 2, on the right side, except from the bottom-right one. In the latter, we can see that, although it might seem counterintuitive, the APs in the same channel (corresponding to the monochromatic edges) allow the improvement of the average vertex interference.

## 4. An Application of Graph Coloring to Channel Assignment in IEEE 802.11 Networks

### 4.1. System Model Using Graphs

#### 4.1.1. Topological Model

As stated above, the problem of assigning channels to an infrastructure Wi-Fi network can be studied as a graph-coloring problem, i.e., channel selection will correspond to the coloring of the vertices of a graph. Strictly speaking, a graph is defined by a tuple (V,E), where *V* is the set of vertices and *E* is the set of edges between those vertices, i.e., E⊆{(u,v)∣u,v∈V}. We used undirected geometric graphs, which means that the nodes in the graph have specific positions in space (i.e., spatial coordinates) and the links between the nodes are bidirectional and symmetric. Our graphs have two different types of vertices: APs and STAs. There are also two types of edges, specifying the type of relationship between their vertices. One type of edge represents the association between the STAs and APs, while the other represents the interferences between the Wi-Fi elements. From now on, we will call them, respectively, *signal* and *interference* edges. Every STA will be connected to its corresponding AP by a signal edge. In addition, two Wi-Fi elements (regardless of being an AP or STA) will be connected by an interference edge if they are associated with different APs. In addition, we define a *cluster* as the set of Wi-Fi elements containing an AP and all its associated STAs. Finally, wireless devices normally do not transmit all the time, so we assumed different activity indexes for the STAs and APs.

**Figure 3:**
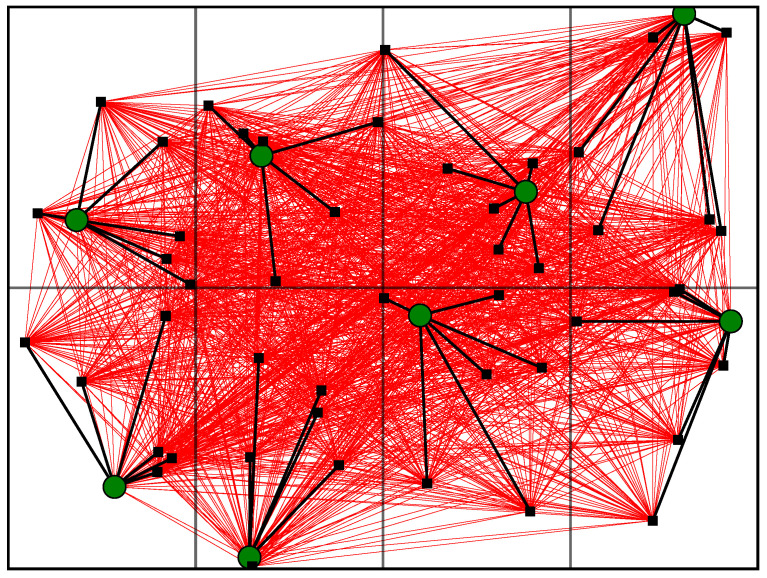
Example of graph for a floor of a building with eight flats and six STAs per AP.

Although we will consider 3D graphs, for the sake of visibility, we show an example of a graph that represents a floor of a building with eight flats, one AP per flat, and six STAs per AP in Figure 3. In this figure, the APs are represented as large green circles, STAs as black squares, signal edges as black lines, and interference edges as red lines.

**Figure 4:**
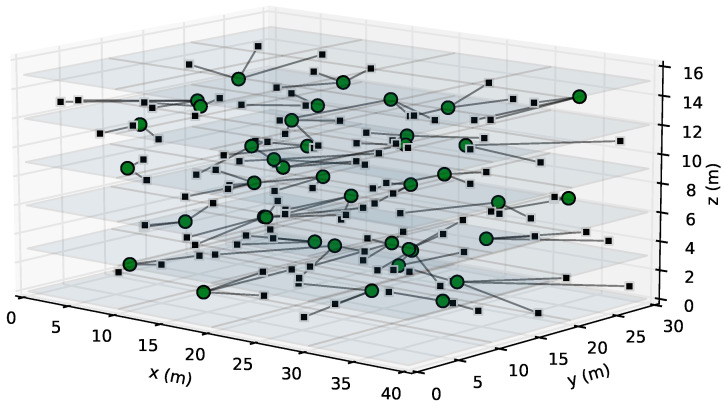
3D realistic uncoordinated Wi-Fi setting under study.

The 3D layouts that we have considered for the experiments consist of a five-floor residential building. This setting is a paradigmatic example of a dense uncoordinated Wi-Fi network. The dimensions of each floor are 40 × 30 × 3 m (length, width, and height, respectively). On each floor, there are eight flats in a four-by-two arrangement. We considered that, in each flat, there is an AP and η STAs (with η ranging from 1 to 10), uniformly distributed in the xy-plane. In the z-axis, the APs and STAs are placed according to a normal distribution with a mean of 1.5 m and a standard deviation of 0.5 m (and bounded to the limits of the floor). Therefore, all the scenarios have 8×5=40 APs while the number of STAs ranges from 40 (for η=1) to 400 (for η=10). Finally, for each setting with a specific value of η, we have five different scenarios, with different random positions for the APs and STAs. Figure 4 shows a graphical representation of the scenario under study with η=3, where, for the sake of visibility, only the signal edges are shown. We can see that the STAs are attached to the AP of the flat they are located in, which is a reasonable assumption.

#### 4.1.2. Propagation, Interferences, SINR, and Throughput Computation

We computed the throughput from the SINR (Signal-to-Interference-plus-Noise Ratio), which can be derived from the geometry of the problem together with the propagation losses and thermal noise. We have used the propagation model defined by the ITU-R in Recommendation P.1238-10 [29]. This defines an indoor transmission loss model, assuming the APs and STAs are inside the same building. The model also takes into account the propagation loss across the different floors of the building. According to that propagation model, the propagation loss for a signal (or interfering signal) with a central frequency of *f* Mhz at a distance *d* meters from its origin can be computed as:

(1) $$L_{total}=20\log_{10}f-28+N\log_{10}d+L_{f}\left(n\right),$$

where *N* is the power loss coefficient and $L_{f}\left(n\right)$ is the floor penetration factor for a signal traversing *n* floors. We will focus on the 2.4 GHz band since it is the most widely used and the more congested and, therefore, it is the most challenging from an engineering point of view. In any case, following Equation (1), it would be straightforward to consider the 5 GHz frequency band instead. The value used for the power loss coefficient *N* has been chosen according to [29,30] because [29] defines, for the 2.4 GHz band, N=28 in residential environments but admits that the signal propagation across the walls increases *N* considerably. For that reason, we have used, according to [30], N=28 when d<16 m and N=38 when d≥16 m. Moreover, we have considered, according to [29], that the signal propagation produces a loss of 10 dB when traversing a floor using concrete. The signal power received in STA or AP *i* from another AP or STA *j* can be computed as:

(2) $$P_{r}^{j\to i}=P_{trx}^{j}+G_{j}+G_{i}-L_{total},$$

where $P_{trx}^{j}$ is the transmission power of source *j* expressed in dBm and $G_{i}$ ($G_{j}$) is the antenna reception (transmission) gain in dB.

Apart from the previous considerations, which are valid for both the desired signal and the interferences, there are some particularities that are relevant when considering interferences. First, to account for the fact that an interfering source will not be continuously transmitting, we included the *activity factor*, denoted as F, which is basically the fraction of time that an interfered device is transmitting. Second, not all the interfering signals will equally affect our signal since the impact will depend on the frequency channel the interfering signal is in. To account for that issue, we introduced factor C. If we would consider a frequency band whose channels were all orthogonal, C would be reduced to a Kronecker Delta function. In fact, this is the trend in Wi-Fi 6 and 7 in the 5 GHz and 6 GHz frequency bands. However, we now focus on the 2.4 GHz band since it is more complex. In this case, there is a partial overlap between the adjacent channels, so we resorted to an empirical definition of C, according to Table 2 [31], where C is a function of the distance (in terms of the frequency difference between the number of channels) δ. Once F and C have been defined, we can easily derive the received power from an interfering signal using:

(3) $$I^{j\to i}=P_{r}^{j\to i}\cdot F\cdot C\left(\delta \right).$$

Using the received power of the desired signal, together with all the interfering signals, we can easily compute SINR as:

(4) $$SINR_{i}=\frac{P_{r}^{\chi \to i}}{\sum_{\forall j\in J}I^{j\to i}+N},$$

where $P_{rx}^{\chi \to i}$ stands for the power of the received desired signal from node χ, J is the set of wireless nodes that emit interfering signals, and *N* is the power of thermal noise (in dBm). The thermal noise can be easily computed as:

(5) $$N=-174+10\log_{10}\left(\Delta f\right),$$

where Δf is the bandwidth of the channel (in Hz).

Finally, we used the SINR to compute the throughput, which depends also on the technology used and its configuration parameters. The most widespread Wi-Fi technology used in the 2.4 GHz frequency band is IEEE 802.11n [32], also known as Wi-Fi 4. More specifically, we have focused on the mandatory Guard Interval (GI) of 800 ns and mandatory 20 MHz channels, although other options could be used [33]. For that configuration, the standard [32] defines eight MCS (Modulation and Coding Schemes), and, depending on the SINR, we will be able to choose a certain MCS. We have used the threshold values for the SINR defined in Table 3 [34] and we will be able to obtain data rates ranging from 6.5 Mbps with MCS0 to 65 Mbbps with MCS7. Summarizing, for each STA, we will compute the SINR using the power of the signal received from its AP and all the interferences and the noise received, and, with that SINR, we can determine the MCS and, therefore, the experienced throughput.

### 4.2. Channel Selection Techniques

There have been many proposals to assign channels in Wi-Fi networks, as we reviewed in Section 1. However, the dominant technique in uncoordinated networks is just configuring the ISP router with the option of *Automatic*, so that it chooses the least congested channel. In better cases, customers use an app on their smartphone to scan the spectrum, determine which is the best channel to use for their Wi-Fi routers, and set up the channel in the routers manually. As this trend in the configuration is expected to continue, we evaluated and compared some of the most simple techniques that customers could use to configure the operation channel of their Wi-Fi networks in a dense uncoordinated 3D Wi-Fi deployment. Although this issue is of paramount importance for the Wi-Fi community, to the best of our knowledge, there is not any study with this comparison.

The techniques we are studying and comparing must fulfill a clear requirement: They must be based on simple information that can be easily gathered by either the STAs or APs. In this sense, we considered two different approaches.

#### 4.2.1. Least Interference Channel Selection (LI) Technique

The first intuition when choosing a channel is to scan the spectrum band measuring the received interference power for each of the available channels. Since interferences fluctuate over time (APs and STAs usually do not have uniform transmission patterns), it is necessary to average some measurements taken over time. Once we have a vector with the measured interference in each of the 11 possible channels, we select the channel with the minimum interference. In the event of a tie between several channels, we choose one of them randomly. If the channel we are already in is in the list of channels with minimum interference, we will stay in the same channel.

#### 4.2.2. Beacon-Based Channel Selection Techniques

To assign channels in a Wi-Fi network, we can also use beacon frames, which are management frames that APs broadcast periodically. So, we can scan the spectrum looking for beacon frames from the other APs to estimate which is the best channel to operate in. There are three different beacon-based techniques that could be easily used by customers to guide the channel assignment process. Two of them, very similar, measure the power of the beacon frames received from the different APs. The third one counts the number of received beacons from the other APs.

For the *Least Beacon Power masked (LBPm)*, we scan the spectrum looking for beacon frames transmitted from other clusters. Moreover, we only consider beacon frames whose receiving power is higher than the receiver sensitivity. After this scan, we aggregate the interference of each beacon frame into the appropriate channel of the spectrum, considering that a beacon frame in a given channel also affects the adjacent channels. With that information, we choose the channel where the power of the different beacon frames received is minimal.

The *Least Beacon Power (LBP)* is very similar to LBPm, but we now restrict the measured power of each beacon frame to the channel where it is received. This operation is very easy to implement, as we know both the channel of the beacon frame and its power in that channel.

The *Least Number of Beacons (LNB)* consists of measuring the number of beacon frames in each channel. To implement this technique, we have to scan the beacon frames that are received with a power higher than the sensitivity of the receiver and count their number in each channel. The chosen channel will be the one with the lowest number of beacon frames detected. In the event of a tie between several channels, we choose one of them randomly (or stay in our current channel if it is one of the tied ones). This technique is similar to the well-known Least Congested Channel Search (LCCS) [2] technique, based on measuring the number of STAs using each channel. As the number of STAs per AP is a constant value in each of the considered scenarios, choosing the channel with the least number of beacon frames (LNB) will be equal to the results obtained by LCCS. However, we have preferred to use the number of beacon frames since it is easier to measure, which is the main requisite of the studied techniques.

#### 4.2.3. Baseline Techniques

Although the main objective is to compare the above-mentioned techniques between them, it is also interesting to compare them with some baselines. In fact, we have considered both lower and upper bounds. In a lower bound, we have included in the results two very simple techniques. The first one is the worst possible choice, corresponding to choosing the same channel for all the APs. Although we have chosen channel six, this specific choice is irrelevant. In the second reference technique, each AP chooses a channel randomly from the set of 11 available channels and, therefore, we will call it *Random*. An interesting feature of these techniques is that they do not require any type of coordination, or even to sense the physical environment.

#### 4.2.4. Optimal Assignment

Finally, in an upper bound, we have considered a centralized optimization approach. More specifically, we have used simulated annealing (SA) to compute the (quasi-)optimal channel assignment distribution for each setting, as we conducted in [13]. SA is a nonlinear optimization technique, and its purpose is to search for better solutions through the state space, evaluating some neighboring states to the current state. After this evaluation, it decides whether to move or not to one of these neighbor states with a finite probability, which depends on the change of performance from the current state to the neighbor, a parameter called *annealing temperature*, so that the probability of moving is higher as the annealing temperature is higher. Moreover, the temperature decreases progressively as the algorithm progresses to guarantee its convergence. In this paper, we have used an implementation of SA, which is an extension to the one in [13]. In this implementation, we generated just one neighbor solution per iteration, by randomly changing the channel where a randomly chosen AP operates. With respect to the probability of movement, we have used the following equation:

(6) $$P_{m}=e^{-\Delta U/\tau },$$

where ΔU is the variation of “utility” (i.e., the throughput loss due to the movement) and τ is the annealing temperature, which starts at one and linearly decreases to zero as the algorithm iterates. The complete algorithm is shown in Algorithm 1 and works as follows:

- We start with a random base solution $S^{b}$, which is basically a random channel assignment for all the APs (1).
- At time *t*, to generate the next candidate solution $S_{t}^{c}$, the optimizer takes the base solution $S_{b}$ and moves to a neighbor solution (2) by choosing a random access point and selecting a new random channel for it.
- When a candidate solution yields a utility loss against the base solution, there will be a probability for the optimizer to “move to it” nonetheless. As shown in Algorithm 1, this probability $P_{m}$ depends on the utility loss associated with the new contract ΔU, and also depends on a parameter known as *annealing temperature* τ (3). Annealing temperature begins at an initial value and linearly decreases to zero throughout successive iterations of the protocol (4).
- If at time *t* the optimizer “moves to” the neighbor solution $S_{t}^{c}$, this solution will be used as the new base solution $S^{b}$ to generate the next neighbor $S_{t+1}^{c}$ (5). Otherwise, the previous $S^{b}$ will be used.
- After a fixed number of iterations, the optimizer returns the final solution, which will be the last base solution (6).

Of course, since SA is a centralized algorithm, it cannot be applied to uncoordinated Wi-Fi networks unless the APs collaborate, which allows for an improvement in performance. Thus, the interest in using SA as a baseline is two-fold, as it represents an upper bound due to its optimal nature and also because it considers that the APs collaborate, sharing their state and accepting the optimal channels that are assigned to them.
**Algorithm 1:** Centralized optimization based on Simulated Annealing **Input:**   *G*: graph model of the Wi-Fi scenario   *T*: maximum number of iterations   $\tau_{0}$: initial annealing temperature **Output:**   *S*: final solution, corresponding to a channel assignment for each AP $U_{0}^{c}=0$ t=1
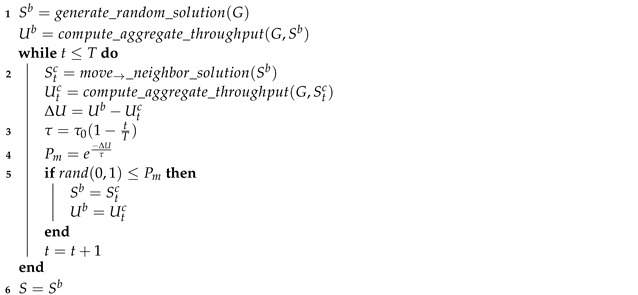



## 5. Experimental Evaluation

This section describes our experimental settings and discusses the results of our experiments using the aforementioned model and techniques. Three different analyses have been performed. First, we compared the performance of the different techniques and then we studied different perspectives for data acquisition for the best different techniques. Finally, the distribution of channels in the selection outcomes is discussed.

### 5.1. Experimental Settings

We have conducted experiments for the five techniques described in Section 4.2 and for all 50 scenarios described in Section 4.1 for a total of 250 experiments. For each experiment, 100 trials were run in the case of the distributed techniques (LI, LBPm, LBP, and LNB). For SA, since its variability is much smaller, we ran 10 trials. For each trial, we recorded the resulting “coloring” (i.e., which channel was finally assigned to each AP) and the resulting throughput obtained.

The proposed techniques are fairly straightforward but, for the sake of reproducibility and statistical significance, we briefly outline here some of the experimental parameters. Since LI, LBPm, LBP, and LNB are asynchronous techniques, we had to “simulate” the asynchronicity. To achieve this, we let each AP change its channel up to 20 times, except if the algorithm converged before. More specifically, in each trial run, there are up to 20 rounds and, in each round, all the APs have the opportunity to change their channels. In each round, the order in which the APs are offered this choice is random. If during a whole round no AP changes its channel, the process has converged and there is no need to obtain the maximum number of 20 rounds. In the case of SA, for each trial, the algorithm was run with a maximum of 3000 iterations and an annealing temperature equal to one.

Regarding the parameters for throughput computation, we have considered a typical transmission power for network elements $P_{trx}=30$ mW, and we have considered simple transmission and reception antennas, with gains $G_{trx}=G_{rx}=0$ dB. Finally, to account for the fact that APs typically transmit for a longer time ratio than STAs and, following that, the interferences produced by the APs are more harmful than those produced by the STAs, we have considered for APs that F=0.5 while for STAs we have considered that F=0.1.

Finally, the implementation of the graphs that model the different realistic uncoordinated Wi-Fi settings has been performed using Python and the *networkx* library [35]. The implementation of the channel selection techniques (both the proposals, baseline, and SA) has been programmed in Python to be easily integrated with the graph model.

### 5.2. Comparison of Techniques

**Figure 5:**
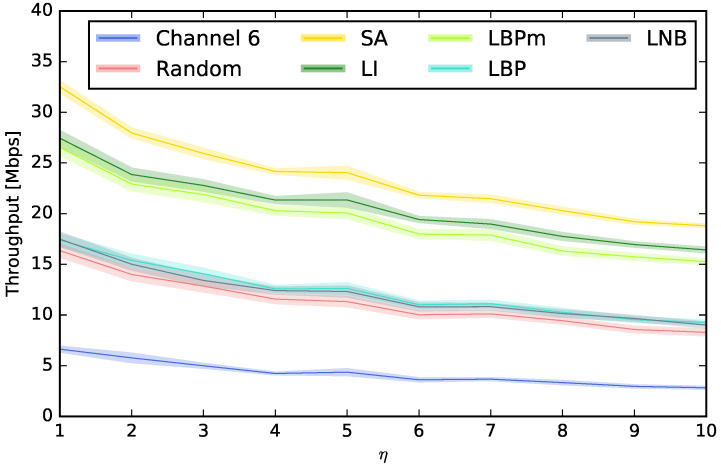
Comparison between channel assignment techniques.

We have first compared all the techniques described in Section 4.2. The purpose is to determine what is the best approach for choosing the Wi-Fi channels for the APs. Figure 5 compares the average downlink throughputs obtained using each technique for the different STA densities. These densities are represented by a range of values of η, i.e., for the different number of STAs per AP, ranging from 1 to 10. This figure also shows the 95% confidence interval. As expected, the worst throughput is obtained when the same channel is used for all the APs, followed by far by the *Random*, LBP, and LNB techniques. From the figure, we can conclude that using LBP or LNB is not recommendable, as their performance is similar to random channel assignment, which is definitely much easier to implement. We can also see that the use of LBPm, and especially LI, is highly recommendable with respect to the rest of the techniques under study. Although the performance of LI and LBPm is fairly similar, LI is slightly better in all cases. Keep in mind that LBPm only considers the power of the beacons received from the APs when this power is higher than the sensitivity of the receivers, while LI considers every interfering signal, especially from the STAs since their number is usually much higher than the number of APs. By analyzing the common aspects of LI and LBPm, we can conclude that including the cochannel interference is a key aspect to achieving better throughput. This conclusion is supported by the fact that the cochannel interference is the only difference between LBPm and LBP, and the performance is significantly better in the first case. Finally, we observed that, although the performance of LI and LBPm is notably higher than the one obtained by LBP, LNB, and *Random*, there is still room for improvement since SA is able to achieve better throughputs. Note that the results obtained by SA must be regarded as a maximum performance bound since its applicability is much more complex in terms of computation cost, and it is also a technique not suited for distributed environments since it uses centralized optimization.

### 5.3. Who Should Decide? Evaluation of Different Perspectives on Channel Selection

**Figure 6:**
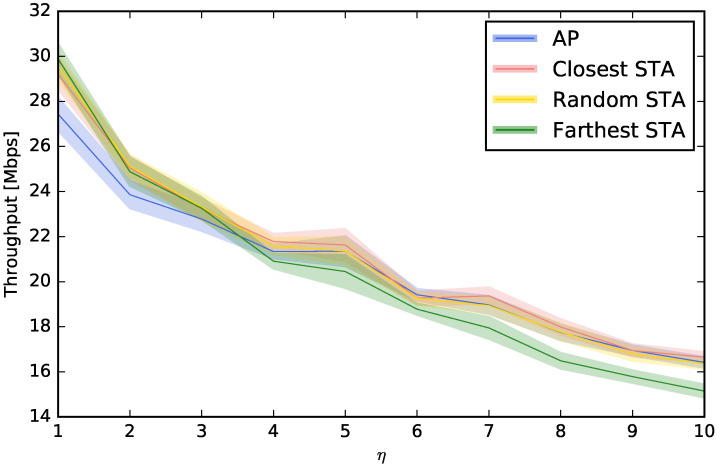
Evaluation of different choices to control the AP using LI.

**Figure 7:**
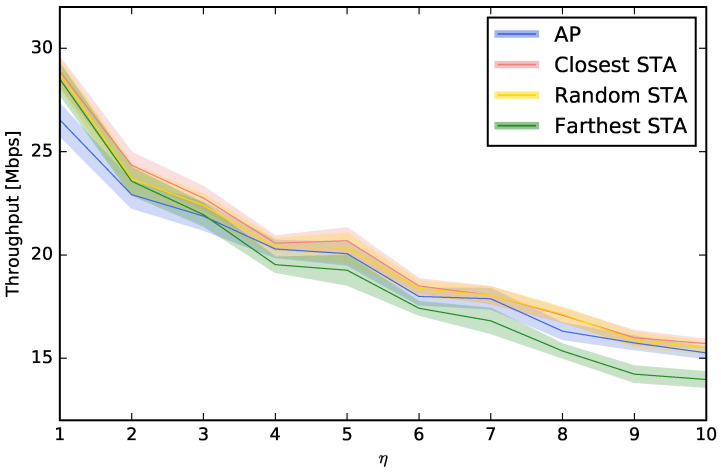
Evaluation of different choices to control the AP using LBPm.

**Figure 8:**
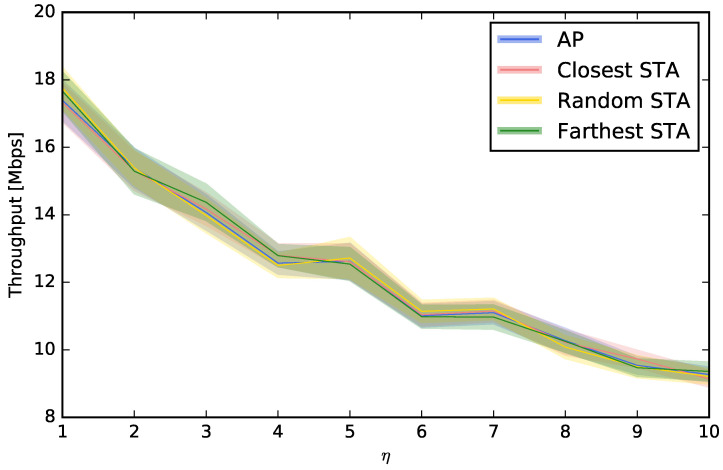
Evaluation of different choices to control the AP using LBP.

**Figure 9:**
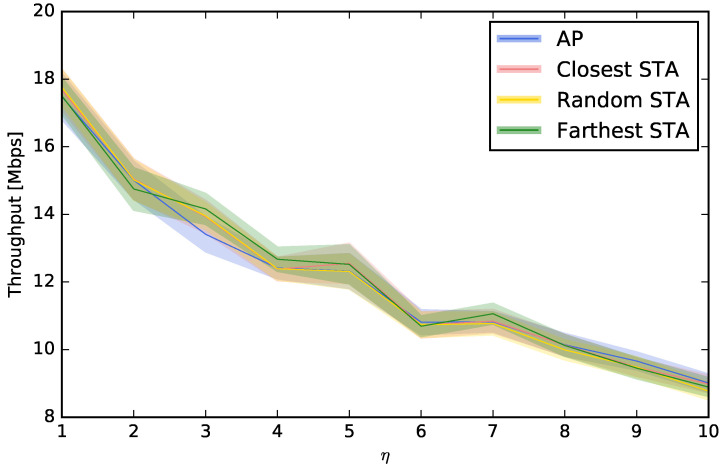
Evaluation of different choices to control the AP using LNB.

In this section, we study where to place the perspective on the channel selection decision. Up to this moment, we have considered that the decision of the channel choice is taken using the “point of view” of the AP, i.e., the interferences, the power of the beacons, or the number of beacons are all measured at the AP. However, this decision can also be taken from the point of view of an STA. For example, a computer or smartphone (STA) can run an application to scan the spectrum looking for beacons or interferences, and this measurement is not the same that the one that would be obtained at the AP. For that reason, we will compare the usual “AP-centric” measurement with others that are more “STA-centric”. More specifically, we will study three options for choosing the STA that scans and configures the AP: (i) the STA that is closest to the AP, (ii) a random STA, and (iii) the STA that is farthest from the AP. Figure 6, Figure 7, Figure 8 and Figure 9 show that, depending on the network density, the best choice may vary. For LBP and LNB, it is not important where to scan the spectrum while for LI and LBPm, in general, it is not recommendable to use the AP when η is low or to use the farthest STA when η is high.

### 5.4. Evaluation of the Use of Channels

**Figure 10:**
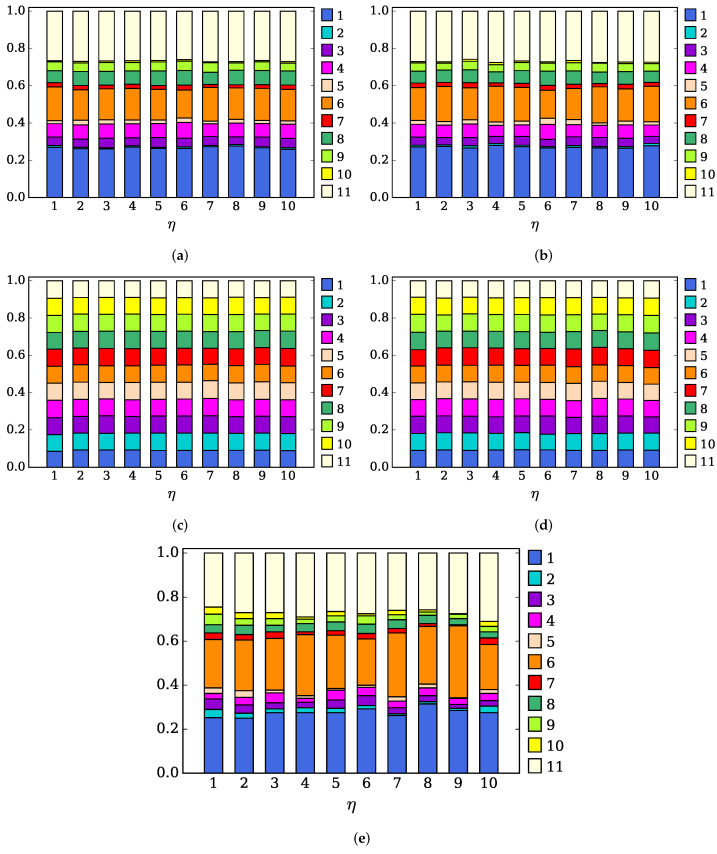
Distribution of channels used under different channel selection techniques. (**a**) LI. (**b**) LBPm. (**c**) LBP. (**d**) LNB. (**e**) SA.

Now, we perform a more in-depth analysis of the distribution of channels used. More specifically, for each value η of the number of STAs per AP, we have run each proposed algorithm 100 times, registering the number of times that each Wi-Fi channel is used by an AP. With that, for each value of η, we have a vector with length 11, where the *k*-th element is the number of repetitions of channel *k* in the solutions obtained. Later, we normalized that vector so that the sum of all its elements equals one, so that it represents the probability of the use of a certain Wi-Fi channel, and we represented that information as a stack in Figure 10, using a different color for each channel. So, the more a Wi-Fi channel is used, the higher the length of the bar of its associated color in the stack. Each figure depicts the results for a particular technique (LI, LBPm, LBP, LNB, and SA), while each column within each figure represents the distribution of the channels for each different value of η under consideration (η∈{1,…,11}).

Figure 10a,b shows the distribution of channels obtained by LI and LBPm. It is significant to note that the distribution of the channels does not depend on the density of users η. Additionally, we can see there is an uneven distribution of channels, i.e., there are certain channels that are used much more often than others. In fact, some channels are very scarcely used. The channels that are most widely used are the three-orthogonal ones, i.e., channels 1, 6, and 11 (especially channels 1 and 11). Channels 4 and 8 are the next channels being favored. Finally, the use of channels 2, 5, 7, and 10 is negligible since they are the closest to the three orthogonal channels. Finally, we can see that LI and LBPm have a natural tendency to choose one of the three orthogonal channels. It is interesting to note how a simple, decentralized channel assignment technique reaches a similar “agreement” to what we normally see in centralized channel assignment proposals [36].

In other words, orthogonal channels are used more often not because the channel assignment techniques are restricted to the use of those orthogonal channels, but because taking into account adjacent channel interference causes a natural trend to use these channels. In fact, many channel assignment proposals restrict their operation to the three orthogonal channels due to the fact that it turns the channel assignment problem into a more tractable one and because it is usually noted that these channels are the most widely used. However, in this paper, we can conclude that the fact that these channels are so widely used is a consequence of taking into account the interferences from the adjacent channels.

Additionally, Figure 10c,d shows that LBP and LNB have a natural trend to use all the available channels evenly, as it would with the *Random* technique. In fact, as we concluded from Figure 5, the performance of LBP and LNB is similar to the *Random* technique.

Finally, Figure 10e shows the use of the channels when using SA, where a similar uneven use of the different channels, such as in the case of LI and LBPm, is shown. This last behavior reinforces the idea that an uneven use of the channels (being the orthogonal channels most widely used) is a consequence of trying to minimize interference.

## 6. Discussion

Channel selection in Wi-Fi networks represents a challenging problem for the theoretical and applied scientific communities. One of the main challenges arises from the cochannel interferences that are produced by the Wi-Fi elements operating in “nearby” channels since the channels partially overlap. In this paper, we showed that graph coloring is a powerful tool to offer a theoretical basis for channel selection. Although classical graph coloring problems cannot be directly applied to channel selection in Wi-Fi networks, we have proposed *spectrum graph coloring* to use graph coloring for channel assignment in IEEE 802.11 networks. In this paper, we used graph coloring to study a very common problem in uncoordinated Wi-Fi settings: How can customers optimize their Wi-Fi operating channel using simple (and easy-to-scan) measurements? These measurements are based on scanning the power of interference signals or the beacon frames that are periodically transmitted by access points (APs). The main strength of this work is the focus on practical and simple measurements, along with the consideration of adjacent channel interference in an indoor 3D propagation model, which has never been studied before.

We compared these techniques with lower-bound baselines (everyone using the same channel or everyone using random channels) and an upper-bound baseline (an optimizer based on simulated annealing). The results show that the best choice is to base the channel selection decision on the measured interference from other APs and stations (STAs), as the resulting throughput is better than measuring the beacon frames (especially if we ignore the cochannel interference). We also concluded that it is worthwhile to make the interference measurement not at the AP (which is where it is measured in most cases) but in the closest STA, as the perceived throughput is slightly better. Finally, we have studied the distribution of the channels that are selected when using the different techniques, showing that the best-performing approaches tend to choose the orthogonal channels much more often than other channels. This is interesting because we see that uncoordinated, simple approaches can obtain outcomes similar to the ones achieved by centralized management. For that reason, we claim that most Wi-Fi networks operate in the orthogonal channels not because APs are restricted to those channels (as many other scientific studies assume) but as a consequence of adjacent channel interference.

Although our experiments yielded satisfactory results, there are further avenues of work in this area. We are interested in developing simple negotiation mechanisms, such as the ones devised in [37], to enable better-distributed cooperation between APs and STAs. We also want to study dynamic models, where the mobility of STAs is introduced and taken into account in the channel assignment mechanism. Finally, we want to study the upcoming Wi-Fi 7 model.

## Figures and Tables

**Table 1:** Related works in the area of channel selection techniques in Wi-Fi.

Reference	Centralized	Distributed	Heuristic	Optimization	Cochannel
[1]	✓			✓	✓
[2]		✓	✓		
[3]	✓		✓		✓
[7]	✓			✓	
[8]	✓			✓	✓
[9]	✓			✓	✓
[10]	✓	✓	✓		✓
[11]	✓		✓		✓
[12]	✓		✓		
[13]	✓			✓	✓
[14]	✓			✓	✓
[15]	✓		✓		✓
[16]	✓		✓		✓
[17]	✓		✓		
[18]		✓		✓	✓
[19]		✓	✓		
[20]		✓	✓		
[21]		✓	✓		
[22]		✓	✓		
This paper		✓	✓		✓

**Table 2:** Spectral overlap between Wi-Fi channels [31].

δ	0	1	2	3	4	5	≥6
C(δ)	1	0.8	0.5	0.2	0.1	0.001	0

**Table 3:** Relationship between MCS, SINR, and throughput in Wi-Fi 4 using 20 MHz channels with mandatory 800 ns GI [32].

MCS Index	Modulation Scheme	Coding Rate	Throughput (Mbit/s)	SINR Range (dB) [34]
0	BPSK	1/2	6.5	(6.8, 7.9)
1	QPSK	1/2	13.0	(7.9, 10.6)
2	QPSK	3/4	19.5	(10.6, 13.0)
3	16-QAM	1/2	26.0	(13.0, 17.0)
4	16-QAM	3/4	39.0	(17.0, 21.8)
5	64-QAM	2/3	52.0	(21.8, 24.7)
6	64-QAM	3/4	58.5	(24.7, 28.1)
7	64-QAM	5/6	65.0	≥28.1

## Data Availability

The graph files and the corresponding EPS figures can be downloaded from https://doi.org/10.5281/zenodo.5703320 (accessed on 17 May 2023). The code (in Python) we have used to evaluate throughput in a given setting is available upon request.

## Abbreviations

The following abbreviations are used in this manuscript:

AP	Access Point
LBP	Least Beacon Power channel selection
LBPm	Least Beacon Power masked channel selection
LCCS	Least Congested Channel Search
LI	Least Interference channel selection
LNB	Least Number of Beacons channel selection
MCS	Modulation and Coding Scheme
SA	Simulated Annealing
SINR	Signal-to-Interference-plus-Noise Ratio
STA	Station
TSC	Threshold Spectrum Coloring
